# DNA studies using atomic force microscopy: capabilities for measurement of short DNA fragments

**DOI:** 10.3389/fmolb.2015.00001

**Published:** 2015-01-29

**Authors:** Dalong Pang, Alain R. Thierry, Anatoly Dritschilo

**Affiliations:** ^1^Department of Radiation Medicine, Georgetown University Medical CenterWashington, DC, USA; ^2^Institut de Recherche en Cancérologie de Montpellier, Institut National de la Santé et de la Recherche Médicale U896Montpellier, France

**Keywords:** AFM, DNA fragments, Ionizing Radiation (IR), cell-free DNA

## Abstract

Short DNA fragments, resulting from ionizing radiation induced DNA double strand breaks (DSBs), or released from cells as a result of physiological processes and circulating in the blood stream, may play important roles in cellular function and potentially in disease diagnosis and early intervention. The size distribution of DNA fragments contribute to knowledge of underlining biological processes. Traditional techniques used in radiation biology for DNA fragment size measurements lack the resolution to quantify short DNA fragments. For the measurement of cell-free circulating DNA (ccfDNA), real time quantitative Polymerase Chain Reaction (q-PCR) provides quantification of DNA fragment sizes, concentration and specific gene mutation. A complementary approach, the imaging-based technique using Atomic Force Microscopy (AFM) provides direct visualization and measurement of individual DNA fragments. In this review, we summarize and discuss the application of AFM-based measurements of DNA fragment sizes. Imaging of broken plasmid DNA, as a result of exposure to ionizing radiation, as well as ccfDNA in clinical specimens offer an innovative approach for studies of short DNA fragments and their biological functions.

## Introduction

**Figure 1:**
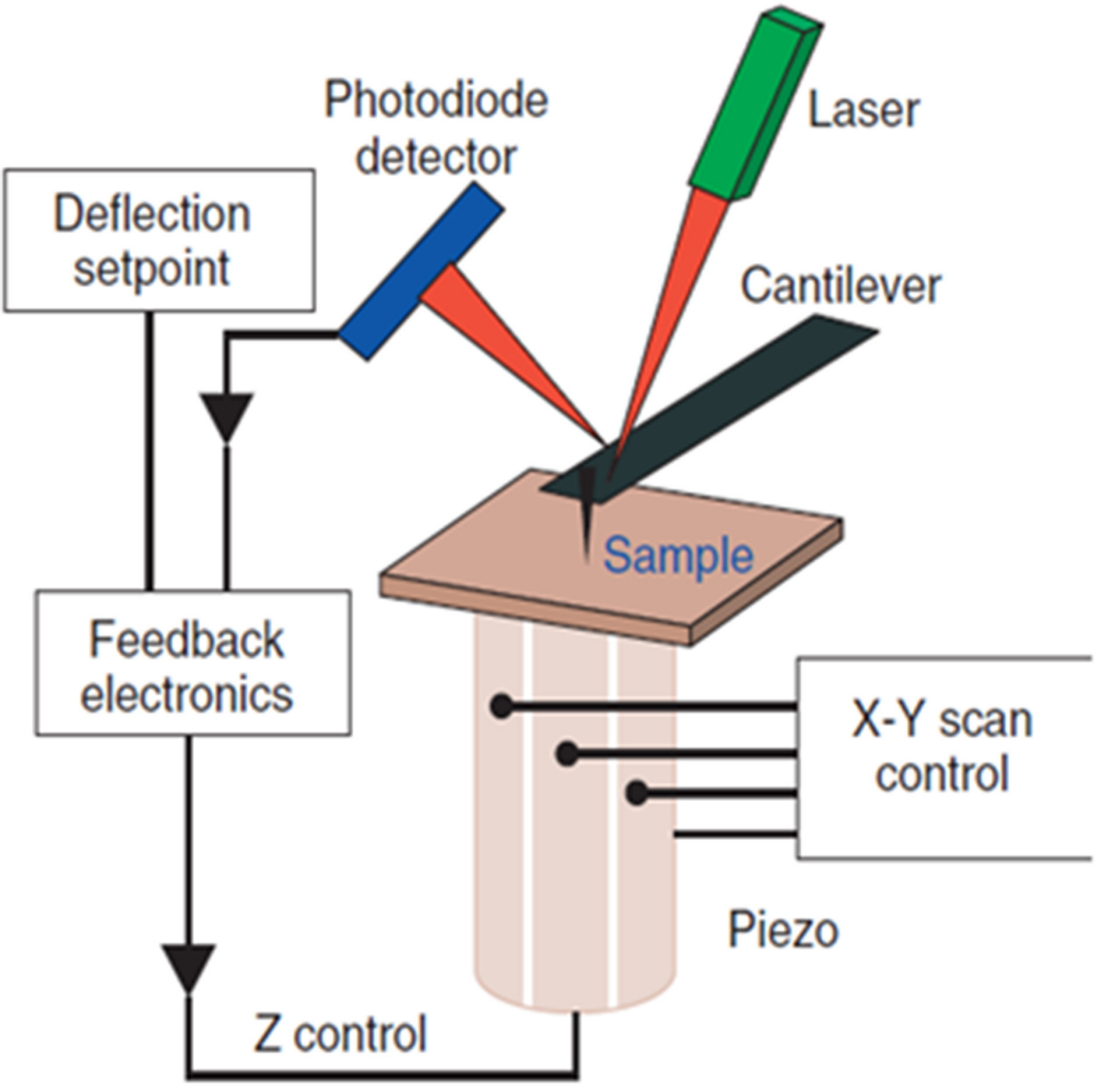
**Schematic diagram of the AFM**. The sample surface topography is sensed by a sharp tip nm in diameter attached to a micro-cantilever. The interaction forces deflect the cantilever; the magnitude of deflection is amplified and converted into a shift of the laser beam spot on the quadruple photodiode detector by the laser beam. The feedback loop controls the vertical position of the piezo so that a constant interaction force between the tip and sample is maintained. The voltage variation used to control the piezo is used to reconstruct the sample topography.

The Atomic Force Microscope (AFM), invented in 1986 by Binnig et al. (1986), employs a nanometer-sized mechanical probe mounted on a micro-cantilever to measure the intermolecular/inter-atomic forces between the atoms on the probe and molecules on a substrate. The repulsive or attractive forces deflect the cantilever and the magnitude of deflection is used to reconstruct the sample topography. The AFM operates by raster-scanning the microcantilever over a sample surface while the sharp probe interacts locally with the atoms of the surface. A pizeo electric platform on which the sample is mounted controls the motion of the sample in the X-, Y-, and Z- (vertical) directions and maintains the desired distance between the probe and the sample surface. The orders of magnitude smaller spring constant of the cantilever (0.01–0.1 N/m) as compared to the vibration spring constant of atoms in a sample (~10 N/m) enables probing of individual atoms of a sample (Lal and John, 1994). Figure 1 depicts the schematic of the AFM detection mechanism.

There are several operational modes employed in AFM. The most commonly used are the contact mode and the non-contact tapping mode. In the contact mode the cantilever tip is brought into physical contact with the sample and scans over the surface. Forces sensed in this mode are the repulsive electrostatic force between the atoms of the tip and those of the sample (Goodman and Garcia, 1991). Due to the relatively strong nature of the force, the contact mode is best used in probing the atomic structure of rigid samples, such as crystals, semiconductors, and metals. In the tapping mode, the cantilever oscillates at its resonant frequency, typically in the range of a few hundred kHz. The oscillation amplitude reduces as the tip approaches the sample surface. Employing a feedback mechanism, the Z (normal to the surface) position of the piezo is adjusted to maintain a preset amplitude while the voltage applied to maintain this amplitude, which directly correlates to the sensed forces, is used to construct sample surface topography. Due to the non-contact nature and high frequency oscillation of the tip, the forces exerted on the sample is much reduced, causing minimum distortion to the sample structure both in the vertical and lateral directions. As a result, Tapping mode has become the preferred mode for imaging soft biological specimens (Hansma et al., 1995, 2000).

Prior to AFM, the two most widely used instruments for atomic and molecular resolution imaging were the Electron Microscope (EM) and the Scanning Tunneling Microscope (STM). The Electron Microscope requires imaging to be conducted in vacuum and cannot be used in liquid environment. Furthermore, the EM requires rather extensive sample preparation, causing potential distortion and damage to soft biological specimens (Hawkes, 1985). The STM is suitable for conducting samples and generally requires a rigid sample surface to obtain high, atomic resolution imaging (Binning et al., 1986). AFM, on the other hand, requires minimum preparation of sample, permits imaging in aqueous solutions, which offers possibilities to examine biological molecules in their native environments and dynamic interactions with other molecules. For these reasons, AFM has become the method of choice for imaging of biological specimens sized below the diffraction limits of light microscopy, in particular, DNA and protein molecules both in air and in solution (Hansma et al., 2004; Sorel et al., 2006; Hamon et al., 2007; Kobayashi et al., 2007; Drew et al., 2010; Cerreta et al., 2012; Esnault et al., 2013; Lyubchenko et al., 2014).

In this paper we focus on a specific AFM application in DNA fragment size measurements, i.e., short DNA fragments generated by ionizing radiation and in quantification of cell-free circulating DNA (ccfDNA) as a potential biomarker for monitoring cancer response to treatment. We propose that AFM can be used effectively to measure individual short DNA fragments of a few tens to a few hundred nm in lengths that are difficult to measure by other techniques, and therefore provide additional information on the biological functions of those DNA fragments.

## AFM investigation of radiation-induced plasmid DNA fragmentation

Ionizing radiation induces numerous DNA lesions, among which DNA double strand breaks (DSBs) have been implicated as the most critical lesion for lethality (Bresler et al., 1984; Barendsen, 1990; Frankenberg-Schwager and Frankenberg, 1990; Mladenov et al., 2013); if unrepaired, a single DSB can lead to cell death (Town et al., 1971; Painter, 1975). As a result, DSBs have been measured extensively both *in vivo* and *in vitro*. In essence, nearly all the techniques for DSB measurements utilize radiation-induced DNA fragmentation to sort irradiated DNA molecules into groups of different conformational profiles. By applying mathematical models, one can calculate the average number of DSBs per unit DNA length, typically Mbp, per unit dose. Traditionally used techniques include neutral gradient sedimentation (Ormerod and Lehmann, 1971; Levin and Hutchinson, 1973), neutral filter elution (Bradley and Kohn, 1979) and gel electrophoresis (Schwartz and Cantor, 1984; Blöcher, 1990). The comet assay is a modified gel electrophoresis technique, permitting measurement of DNA fragments in a single cell (Olive, 2002). An exception to these techniques is the γ-H2AX assay, which utilizes the florescence of the phosphrolation of H2AX histone when a DSB is created (Pilch et al., 2003; Sedelnikova et al., 2003). While highly sensitive, capable of measuring a single DSB, the γ-H2AX assay is not suited to measuring DNA fragment size distributions.

Common to all the fragmentation-based DSB measurement techniques is the limited resolution in fragment size quantification, making it challenging to measure short DNA fragments. Gel electrophoresis, which yields the highest resolution and is the most widely used, separates DNA fragments into bands of different lengths in a gel lane. DNA size markers are referenced for measuring DNA fragments above a few kbp in size (Rydberg, 1996).

In contrast to the traditional techniques, AFM offers an imaging-based, single-molecule method. DNA molecules following exposure to ionizing irradiation can be imaged in air or in aqueous solution on atomically flat substrates. Individual DNA fragments as short as a few nanometers can be measured. A size distribution profile is then constructed summing the numbers of fragments according to their sizes in pre-specified size ranges. The average number of DSBs per DNA molecule, and more informatively, the number of DSBs in various length bins can be determined using simple arithmetic formula, leading to the determination of the spatial DSB distribution on a DNA molecule (Pang et al., 1998, 2005; Psonka-Antonczyk et al., 2009). Such a unique capability for the determination of DSB spatial distribution is especially suited to studying formation of DNA fragments after exposure of DNA to radiations of various linear energy transfer (LET); in particular, high-LET radiations, which yield larger numbers of short DNA fragments (Rydberg, 1996; Pang et al., 1998).

High-LET radiations have produced cell killings 3–20 times greater than that observed following low-LET radiations (Prise et al., 1994; Hall and Giaccia, 2011). However, experimental results have generally shown DSBs comparable to or only slightly higher following irradiation with particles (Jenner et al., 1992; Lett, 1992; Schafer et al., 1994; Taucher-Scholz et al., 1995), a finding inconsistent with the expectation of DSB as the most lethal lesion. The Multiply Damaged Site model, proposed by J.F. Ward, hypothesized the formation of clustered DNA lesions including DSBs, which results in a large number of short DNA fragments, as an important determinant of cell killing (Ward, 1994). Subsequently, Monte Carlo simulation and other biological measurements have validated such clustered DSBs (Nikjoo et al., 1994; Pogozelski et al., 1999). Short DNA fragments recognized to result from clustered DSBs were not properly measured due to sensitivity limitations of the previously available techniques.

**Figure 2:**
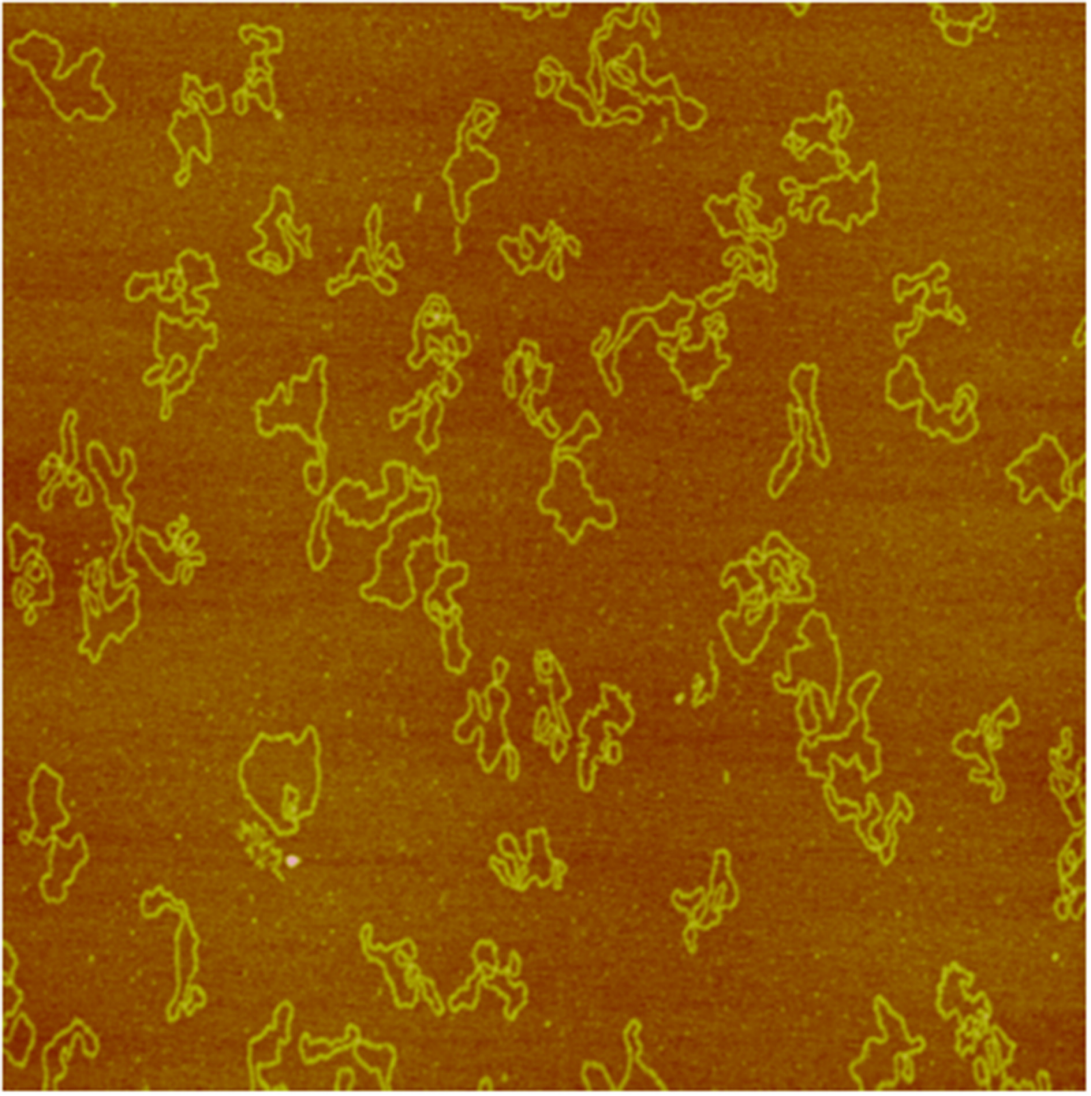
**Image of puC19 plasmid DNA molecules acquired with a NanoScope IIIa AFM in tapping mode in air (so were Figures 3, 4A,B)**. The size of the image is 2 × 2 μm^2^.

**Figure 3:**
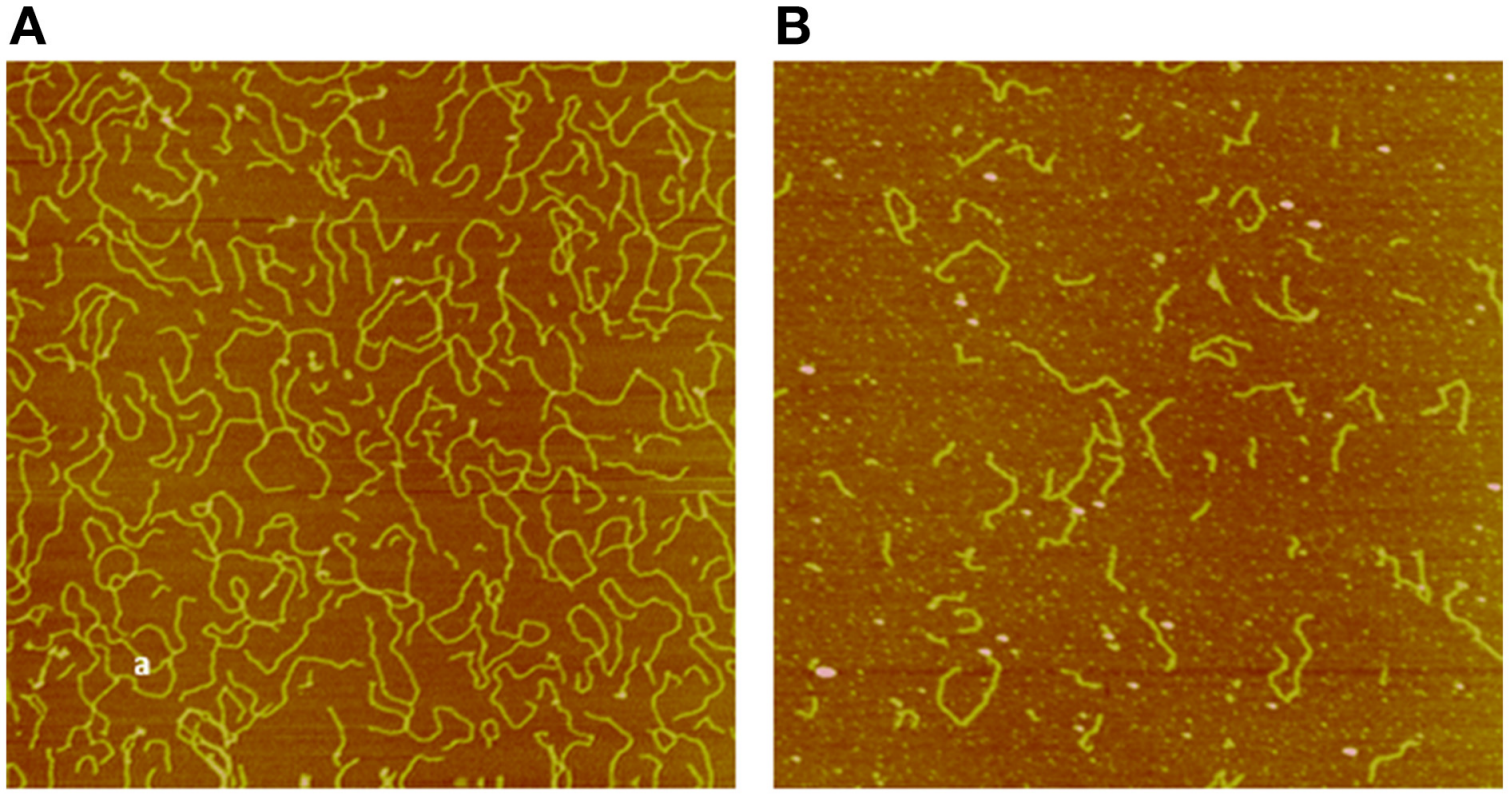
**Sample AFM images of pUC19 DNA exposed to 5 kGy dose of electron (A) and neutron (B) irradiation**. The size of the image is 2 × 2 μm^2^. Data were adapted from Pang et al. (1998).

Applying AFM, we investigated pUC19 DNA fragmentation after exposure to electron and neutron irradiation to various doses, measured the DNA fragment sizes individually, and constructed DNA fragment size distributions for each irradiated sample (Pang et al., 1998). We showed that neutrons, as a high-LET radiation, induces substantially more short DNA fragments than the low-LET electrons, demonstrating clustered DNA damage induced by high-LET radiations. This application of AFM to DNA damage investigation by radiation established the potential utility of AFM in individual DNA fragment size measurements; in particular, short DNA fragments resulting from high-LET radiations not measured with the other techniques. As an illustrative example we show in Figures 2, 3, respectively, sample AFM images of pUC19 DNA before and after exposure to electron and neutron irradiation to demonstrate the capability of AFM in measuring individual DNA fragments.

Measurement of the lengths of individual DNA fragments has permitted easy quantification of the number of DSBs incurred on a DNA molecule. Using the mathematical formulation outlined in reference (Pang et al., 1998), one can estimate the average number of DSBs per DNA molecule. More importantly, the average number of DSBs per DNA that has been broken by radiation can be similarly determined. Furthermore, in addition to the average number of DSBs per DNA, the spatial distribution of the DSBs along a DNA molecule can be determined (Pang et al., 2005). This information provides a sensitive, simple, and straightforward means for the delineation of the radiation damage effects in plasmid DNA by radiations of various LET. The gel electrophoresis technique, which samples only large DNA fragments in smeared bands, lacks such resolution to quantify short DNA fragments, leading to the observed similarity in DSBs by radiations of different LET (Prise et al., 1994).

Other investigators have subsequently applied AFM for further studies of radiation induced DNA damage (Murakami et al., 2000; Brons et al., 2004; Psonka et al., 2005; Brezeanu et al., 2007; Elsässer et al., 2008; Ke et al., 2008; Gudowska-Nowak et al., 2009; Lee et al., 2009; González et al., 2012). Of particular relevance to measurement of short DNA fragments by high-LET radiations, Psonka-Antonczyk et al. (2009) investigated DNA breakage by Ni ions and observed a reduced average DNA fragment length after 340-Gy irradiation when compared to X-ray irradiations. The minimum DNA fragment size they measured with AFM was 100 nm. Brezeanu et al. (2007) studied carbon-ion induced DNA fragmentation on ϕX174 plasmid DNA and reported a significant increase of short DNA less than 250 nm and the number of DSB per broken DNA after carbon ion irradiation when compared to X-rays, a result consistent with our earlier findings with neutron irradiation. Elsässer et al. (2008) further analyzed DNA fragmentation patterns by carbon, nickel, and uranium ions. They observed increased short DNA fragments generation as the LET of radiation increases, and compared DNA fragment size distributions with model predictions. They reported a minimum measurable fragment length of 150 bp (50 nm). These studies have validated AFM as a viable method for the measurement of radiation-induced DNA fragmentation. In majority of these studies, other than a report by Brons et al. (2004), using a contact mode in liquid, tapping mode in air was employed for AFM imaging. Sample preparation techniques for AFM imaging were largely similar, consisting of depositing a few μl of DNA solution in MgCl_2_ and Hepes buffer on freshly cleaved mica surface, rinsed with distilled and de-ionized water, and dried in a gentle flow of nitrogen gas.

Here, we briefly discuss some relevant technical aspects of AFM as a DNA fragment size measurement technique. (1) As is well known, the size of a biological molecule measured with an AFM is the resultant convolution of the size of the AFM tip and that of the molecule (Esnault et al., 2013). Standard AFM tips have a radius of ~10 nm, setting the lower limit of measurable DNA fragment sizes. High resolution images using specially fabricated tips, such as the ultra-sharp tip have been reported (Santos et al., 2013; Mazur and Maaloum, 2014) and structures such as the major and minor grooves (2–3 nm) of a DNA molecule have been demonstrated (Ido et al., 2013). However, even under optimal operational conditions (optimized AFM operational parameters and favorable environmental factors (humidity, noise and vibration etc.), it is difficult to measure DNA fragments only a few nm long, due to difficulties in DNA fragment identification of such short lengths. Consequently, DNA fragment measurements were restricted to 20 nm or greater in our own studies, and 150 bp (50 nm) in Elsässer et al. (2008) and Gudowska-Nowak et al. (2009) studies. (2) To reliably identify short DNA fragments and obtain sufficient quality images, clean samples with minimum amount of other biological components, such as cellular proteins, need to be prepared. Such high purity DNA is obtained using plasmid DNA; therefore, measurements of chromosomal DNA fragments generated in cells will require the development of DNA techniques suitable for AFM imaging. (3) To generate enough short DNA fragments to permit sampling in a reasonable number of AFM images, investigators have used high radiation doses in the kGy range to irradiate and break the DNA molecules, raising the obvious question of biological relevance, where typical therapeutic radiation doses are only a few Gy (Pang et al., 1998; González et al., 2012).

The 10^6^ orders of magnitudes larger target size of genomic DNA should reduce the required radiation dose to biologically relevant levels; however, extraction of short DNA fragments from cells remains a challenge (Lea, 1955; Dolezel et al., 2003). Until this challenge is overcome, use of AFM for DNA fragment size measurements will be limited to non-cellular environment, as evidenced by the lack of publications for AFM imaging of irradiated chromosomal DNA.

In summary, as an imaging technique of nm resolution, AFM is well suited to measuring DNA fragments a few tens to a few hundred nm in lengths resulting from ionizing radiation induced DNA DSBs. To obtain images of sufficient quality, the DNA samples should be clean, devoid of other biological components which may interfere with DNA fragments identification. The target DNA should have well-defined uniform size for DSB quantification based on DNA fragment size measurements. In addition, to obtain sufficient number of fragments to yield statistically meaningful results, radiation doses need to be sufficiently high, typically in kGy. Due to these requirements, AFM is presently useful mostly for *in vitro* DNA fragment size measurements. Nevertheless, the information obtained can be valuable for the studies of physical mechanisms of radiation induced DNA damage, aid in theoretical modeling, such as Monte Carlo simulation and microdosimetry studies, as well as investigation of scavenging or radiation protection effects of various biochemical agents.

## AFM application in cell-free circulating DNA research

Cells, normal or malignant, actively shed DNA fragments into the blood stream during their growth or proliferation (Mead et al., 2011; Schwarzenbach et al., 2011; Mouliere and Thierry, 2012). The DNA fragments (ccfDNA) carry similar genetic or mutational information as that in the cells from which they are originated (Stroun et al., 2001; Diehl et al., 2008; Jung et al., 2010; Mouliere et al., 2013). It was discovered that the concentration of the DNA fragments is significantly enhanced in cancer patients as compared to healthy individuals (Herrera et al., 2005; Chun et al., 2006; Kamat et al., 2010; Mead et al., 2011), and the concentration may be correlated to tumor size and stage (Bremnes et al., 2005). Research has also been conducted to investigate the ccfDNA variation in response to chemo- or radiation therapy (Gormally et al., 2004; Kamat et al., 2006). Successful treatment results in reduced ccfDNA concentration, whereas tumor progression is associated with increased ccfDNA production (Marzese et al., 2013; Nygaard et al., 2013; Oxnard et al., 2014).

Quantification of ccfDNA concentration is primarily carried out using real time quantitative Polymerase Chain Reaction (q-PCR) technique, which permits amplification of selected ccfDNA sizes and provides relative concentration ratios of certain size groups (Wang et al., 2003). Such capability has lead to determination of the Integrity Index for comparison of DNA sizes, in addition to concentration measurement (Holdenrieder et al., 2008).

Presently, a challenge facing the q-PCR technique is the large variation and conflicting results obtained by different laboratories in measuring ccfDNA concentrations for cancerous vs. non-cancerous samples and in assessing diagnostic and prognostic potential in monitoring cancer treatment (Jung et al., 2010). Use of ccfDNA as a reliable biomarker in cancer management requires further research for improved reproducibility.

AFM, with its capability to measure individual DNA fragments, especially short DNA fragments, is well-suited to measuring DNA fragments extracted from the blood. These fragments are usually short in size and clean of other cellular or blood components. ccfDNA fragment distribution was studied by using nested q-PCR systems and showed that most of ccfDNA fragments are below 100 bp (Mouliere et al., 2011). Unlike the q-PCR method, which selectively amplifies certain sizes of DNA, AFM offers a means to obtain complete DNA fragment size distributions. Taken together with ccfDNA concentration, information on the stage or response of cancer cells to treatments can be obtained. Treatment failure which results in continued growth of cancer cells may be manifested by respective ccfDNA profiles.

**Figure 4:**
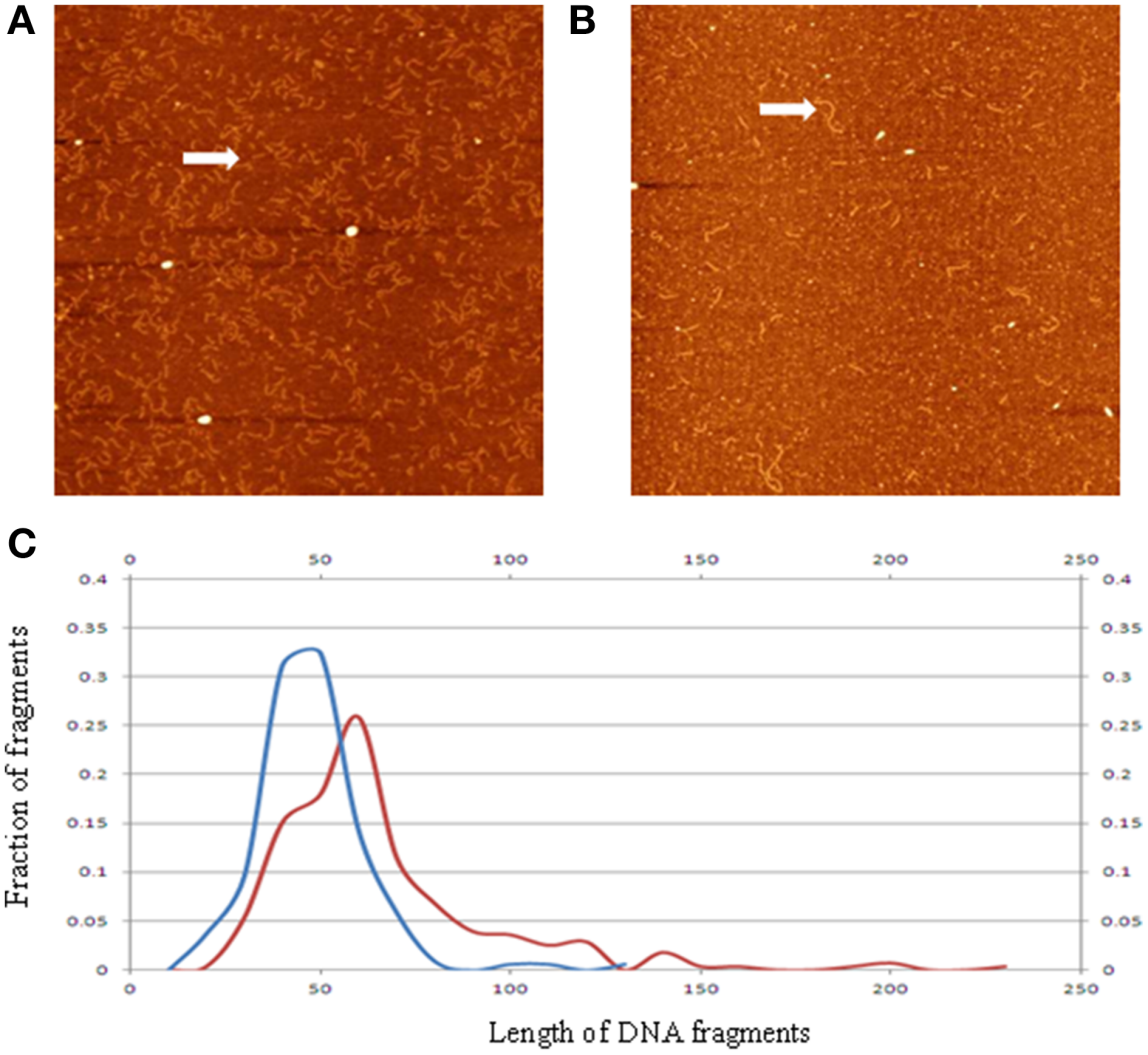
**Representative AFM image of ccfDNA extracted from a patient with cancer (A), and that from a healthy control (B)**. Panel **(C)** displays the averaged ccfDNA fragment size distributions for six cancerous samples (blue curve), and healthy samples (red curve). The vertical axis is percentage of number of DNA fragments; the horizontal axis is length in nm. Arrows in the images highlight sample DNA fragments of different sizes (reconstructed from data in Mouliere et al., 2014).

Applying the AFM technique, we analyzed ccfDNA fragments extracted from patients with colorectal cancers and healthy controls (Mouliere et al., 2014). As shown in Figure 4, the measured DNA fragment size distribution for the cancerous samples exhibit a shift toward the shorter sizes than the healthy controls. This confirmation of earlier observation from q-PCR analysis by another method is critical since it was previously hypothesized that the lower ccfDNA fragment size, as determined by gel electrophoresis, was ~180 bp which is the size of DNA length in a mononucleosome. Statistical analyses of the measured profiles can help identify quantifiable parameters to differentiate ccfDNA samples extracted at different stages of cancer treatments.

## Summary and future prospective

Short DNA fragments, generated by ionizing radiation or shed into the blood stream from cells, are of biological significance. However, measurements of short DNA fragments have been challenging due to limitations in traditional techniques. AFM, as a nanometer resolution microscope, can be used effectively for the measurement of individual short DNA fragments. The measured fragmentation size distributions offer valuable experimental data for the investigation of the physical mechanisms of DNA damage by radiations of different LET; in particular, high-LET radiations. In addition, they are used as input data for Monte Carlo modeling of DNA damage by ionizing radiation. Another potential utility of the AFM is for the measurement of short ccfDNA fragments circulating in the blood. The measured ccfDNA fragment size distributions may be used as a potential biomarker for disease diagnosis and prognostic indicator for treatment response. The examples discussed in this paper demonstrate that AFM can be a valuable technical supplement for DNA size measurements.

The majority of AFM measured DNA data discussed in this paper were specific to radiation-induced DNA fragmentation or ccfDNA, which were acquired with earlier generations of AFM using operational techniques accessible to general AFM users. Since then many new AFM models and more advanced techniques have been developed permitting much finer DNA structure to be probed, for example, the DNA major and minor grooves (Cerreta et al., 2012; Ido et al., 2013; Mazur and Maaloum, 2014). It is predictable that smaller DNA fragment sizes than discussed in this review can be measured. It may be possible to even probe radiation-induced DNA structural damage, such as that to the phosphate group and base damage, opening up boarder area of AFM application in this particular field.

### Conflict of interest statement

The authors declare that the research was conducted in the absence of any commercial or financial relationships that could be construed as a potential conflict of interest.
